# Evaluating institutional climate: Welcomeness, belonging, and well‐being in dental education

**DOI:** 10.1002/jdd.13796

**Published:** 2025-05-27

**Authors:** Tawana K. Ware, Felicia L. Tucker‐Lively, Rosa Chaviano‐Moran, Cherae Farmer‐Dixon, Riki Gottlieb

**Affiliations:** ^1^ Private Practice Indiana University School of Dentistry Indianapolis Indiana USA; ^2^ Academy for Advancing Leadership Atlanta Georgia USA; ^3^ Rutgers University School of Dental Medicine Newark New Jersey USA; ^4^ Meharry Medical College Nashville Tennessee USA; ^5^ Tufts University School of Dental Medicine Boston Massachusetts USA

**Keywords:** climate assessment, dental education, diversity, educational environment, equity, faculty development, inclusion, institutional climate, mental health

## Abstract

**Purpose:**

As part of the American Dental Education Association's (ADEA) comprehensive climate assessment initiative, this study examines institutional climate in dental education programs, focusing specifically on welcomeness, sense of belonging, and well‐being among faculty, staff, students, and administrators.

**Methods:**

Drawing from ADEA's 2022 Climate Study dataset, this analysis focused on 25 selected items from responses of 8485 students, 1994 staff, 3393 faculty, 293 administrators, and 395 individuals with both administrative and faculty roles. Perceptions across three domains: welcomeness, belonging, and well‐being were assessed using a five‐point Likert scale (1 = strongly disagree to 5 = strongly agree).

**Results:**

On the five‐point scale, faculty and administrators reported higher welcomeness (mean = 4.19/5.0, standard deviation [SD] = 0.970) compared to students (mean = 4.06/5.0, SD = 0.924) and staff (mean = 3.99/5.0, SD = 0.930). Sense of belonging varied, with administrators holding faculty roles reporting the highest levels (mean = 4.10/5.0, SD = 0.787), while staff reported the lowest (mean = 3.58/5.0, SD = 0.939). Well‐being measures followed similar patterns, with administrators holding faculty roles reporting the highest personal well‐being (mean = 4.01/5.0, SD = 0.778), social well‐being (mean = 4.11/5.0, SD = 0.638), and climate‐related well‐being (mean = 4.17/5.0, SD = 0.777).

**Conclusions:**

Findings suggest that administrators feel the most comfortable, respected, and supported in the academic environment. Age and experience have minimal impact on feelings of welcome and well‐being. The study reveals significant disparities in perceptions of welcomeness, belonging, and well‐being across different roles in dental education programs. These results highlight the need for targeted initiatives and supportive practices to foster a more inclusive and supportive educational environment for all community members.

## 1 INTRODUCTION

In dental education, institutional climate significantly influences the experiences of students, faculty, and staff. In educational research, institutional climate encompasses the shared perceptions and experiences that shape organizational life. This construct differs from the physical environment or general atmosphere, focusing specifically on how community members experience and interpret their institutional context. Feeling welcome, having a sense of belonging, and overall well‐being are crucial elements of a positive educational environment. These factors impact academic performance, professional development, and mental health.^1^ ^,^ ^2^

Earlier studies have explored individual aspects of institutional climate in dental education.^1^, ^2^, ^3^ Ester et al. examined climate studies from deans' perspectives,^1^ while Radford and Hellyer investigated belongingness in undergraduate dental education.^2^ However, a comprehensive assessment of welcomeness, belonging, and well‐being across different roles has been lacking in dental education literature.^3^

The American Dental Education Association's (ADEA's) 2022 Climate Study addressed this knowledge gap through a comprehensive assessment of dental schools and allied dental education programs across the United States and Canada. From this extensive dataset, our analysis specifically examines three critical dimensions of institutional climate: welcomeness, sense of belonging, and well‐being among administrators, faculty, staff, and students. This focused investigation builds on previous work highlighting the importance of belongingness in student development and well‐being in dental education.^2^ ^,^ ^3^ By analyzing responses across various roles within dental education institutions, our study provides insights into how different groups experience the educational environment. This approach offers a more nuanced understanding of institutional climate than previously available in dental education literature. While dental education programs operate within larger institutional frameworks, understanding program‐specific climate provides crucial insights for targeted improvements.

Building on this foundation, the current study aims to: (1) assess perceptions of welcomeness, sense of belonging, and well‐being among administrators, faculty, staff, and students in dental education programs. (2) Identify disparities in these perceptions across different roles within institutions. (3) Provide insights for improving institutional climate in dental education. These objectives address a critical gap in current literature by offering a more holistic view of institutional climate across various roles in dental education.

## 2 MATERIALS AND METHODS

### 2.1 Study context and data source

This analysis examines climate perceptions within dental education through data obtained from the ADEA's 2022 Climate Study. This comprehensive assessment was conducted over a 4‐month period from January to April 2022, targeting dental schools and allied dental education programs across the United States and Canada. The study protocol received ethical approval from Adverra, Inc. (Protocol number Pro00057545), with ADEA overseeing data collection and implementing robust security measures including data encryption and password protection. Participation was voluntary and no compensation was provided to respondents.

### 2.2 Research questions

Our analytical framework was guided by three primary research questions that explored the multifaceted nature of climate perceptions in dental education. First, we investigated how perceptions of welcomeness, belonging, and well‐being varied among different institutional roles, including students, staff, faculty, and administrators. Second, we examined the relationships between demographic factors, particularly age and years of experience, and climate perceptions. Finally, we analyzed how institutional characteristics correlated with reported levels of welcomeness, belonging, and well‐being among participants.

### 2.3 Survey structure and variable selection

The ADEA's Climate Survey instrument was structured in three distinct sections, progressing from institutional context to individual experiences. The first section captured educational background information through seven questions addressing program type and location. The second and most extensive section comprised 90 questions (questions 8‒98) focused on institutional climate measures. The final section gathered demographic information through eight questions (questions 99‒106).

From this comprehensive instrument, we conducted a focused analysis of 25 items specifically chosen for their direct relevance to our research questions. Our selection criteria prioritized items that provided clear measures of welcomeness (eight items), belonging (nine items), and well‐being (eight items) within the dental education environment. Selection criteria included: (1) direct measurement of target constructs, (2) applicability across all role groups, and (3) demonstrated reliability in previous climate research.

### 2.4 Measures and index construction

The instrument was comprised of Likert style questions with a five‐point answer scale ranging from 1 = disagree strongly, 2 = disagree, 3 = neither disagree nor agree, 4 = agree to 5 = agree strongly. In addition to the Likert style questions described, additional sets of questions with a “yes”/“no” answer format were included in the analyses.

We developed three primary indices to capture distinct aspects of the institutional climate. The Welcomeness Indices measured institutional inclusivity through three components: the “all welcome” index, averaging questions 18‒20 to assess institutional welcome, respect, and support; the “students welcome” index, derived from questions 36‒38 focusing on student‐specific experiences; and the “DEI committee welcome” score, summing affirmative responses to questions 53‒55 regarding diversity, equity, and inclusion (DEI) initiative awareness. The belonging indices comprised two measures: the “sense of belonging” index, combining questions 51, 52, 12‒14, and 16‒17 to assess community connection, and the “no sense of belonging” score, aggregating exclusion experiences from questions 92‒95. The well‐being indices included three dimensions: personal well‐being (questions 9, 15, 22, 23), social well‐being (questions 24‒26, 45, 50), and climate‐related well‐being (questions 10a and 11), each capturing distinct aspects of participant wellness within the institutional environment.

### 2.5 Statistical analysis

Our analytical approach employed SPSS (version 29) to conduct a systematic examination of the data, which ADEA provided in CSV format with a comprehensive codebook. The analysis began with descriptive statistics to characterize the sample and index scores, followed by univariate analyses of variance to examine differences between respondent groups. We investigated relationships between indices and demographic variables using Pearson correlation coefficients. To maintain statistical rigor across multiple comparisons, we applied the Bonferroni correction,^4^ setting the significance threshold at *p* < 0.001.

### 2.6 Sample characteristics

The analytical sample represented a diverse cross‐section of dental education, comprising 8485 students, 1994 staff members, 3393 faculty members, 293 administrators, and 395 individuals holding both administrative and faculty roles. This distribution provided robust representation across institutional roles, enabling meaningful comparisons of climate perceptions. Detailed demographic characteristics of the sample are presented in Table 1. While the survey collected data on program types (dental school/allied program), geographic location, and institutional characteristics (public/private), our evaluation focused on role‐based differences to identify patterns transcending institutional variation.

**TABLE 1:** Background and work characteristics by respondent type.

	Students	Staff	Faculty	Admin	Admin and faculty	*p*‐value
**Background characteristics**						
Number of respondents	8485	1994	3393	293	395	<0.001
Gender:						<0.001
Women	74.2%	82.9%	61.1%	70.1%	64.7%	
Men	24.9%	15.4%	38.0%	29.5%	34.7%	
Other	0.6%	1.1%	0.4%	0.0%	0.0%	
Prefer not to describe	0.3%	0.7%	0.6%	0.4%	0.6%	
Self‐reported race:						<0.001
American Indian	2.8%	3.1%	1.3%	1.2%	1.4%	
First Nation, Indigenous	0.1%	0.3%	0.2%	0.0%	0.3%	
Middle Eastern, Arab or North African	5.6%	1.4%	3.6%	1.6%	3.4%	
African American/Canadian	7.5%	10.6%	4.3%	13.3%	5.6%	
Hispanic/Latinx	14.0%	15.0%	9.5%	9.8%	10.2%	
Native Hawaiian/Pacific Islander	0.4%	0.5%	0.1%	0.0%	0.8%	
Asian American	19.4%	7.3%	11.5%	5.9%	8.2%	
European American	50.2%	61.8%	69.4%	68.2%	70.1%	
Age:						<0.001
Mean	26.80	47.00	54.06	52.71	56.45	
Standard deviation	4.966	12.661	13.304	11.647	10.490	
Range	18–59	20–84	23–89	25–79	23–80	
Self‐reported diverse ability:						<0.001
No	83.8%	86.9%	90.5%	92.3%	90.9%	
Yes	16.2%	13.1%	9.5%	7.7%	9.1%	
**Work characteristics**						
Type of academic program:						<0.001
Dental school	58.8%	87.4%	68.4%	85.5%	75.2%	
Allied dental program	41.2%	12.6%	31.6%	14.5%	24.8%	
Employment status:						<0.001
Full‐time paid	0%	93.2%	62.4%	97.7%	96.7%	
Part‐time paid	0%	6.8%	37.6%	2.3%	3.3%	
Years in dental education:						<0.001
Mean	n/a	12.87	18.74	16.65	22.55	
Standard deviation		11.791	14.113	12.395	11.663	
Range		0–62	0–71	0–49	0–56	
Years employed in current dental school/allied program:	n/a					<0.001
Mean		8.84	12.06	12.31	14.62	
Standard deviation		9.028	11.422	10.314	11.083	
Range		0–67	0–68	0–46	0–54	

Abbreviation: Admin, Administrators.

This analytical approach revealed several key findings about climate perceptions in dental education, demonstrating systematic differences in how various groups experience their educational environment. Below, we present these findings, beginning with demographic characteristics and moving through our three primary measures of institutional climate: welcomeness, belonging, and well‐being.

## 3 RESULTS

Analysis of the ADEA's Climate Study data revealed systematic differences in how various groups experience the dental education environment. The study encompassed a diverse sample of 14,560 respondents from dental education programs throughout the United States and Canada. Students comprised the majority of respondents (8485; 58.3%), followed by faculty (3393; 23.3%), staff (1994; 13.7%), administrators (293; 2.0%), and individuals holding both administrative and faculty positions (395; 2.7%). Gender distribution showed notable variation across these groups, with women representing the majority among students (74.2%) and staff (82.9%), while faculty and administrative positions displayed a more balanced gender composition (see Table 1 for complete demographic characteristics).

### 3.1 Dimensions of institutional climate experience

Our examination revealed consistent patterns across the core dimensions of institutional climate: feeling welcome, sense of belonging, and well‐being; with clear role‐based variations emerging from the data. Faculty and administrators reported notably higher welcomeness scores (mean = 4.19, standard deviation [SD] = 0.970) compared to students (mean = 4.06, SD = 0.924) and staff (mean = 3.99, SD = 0.930). This pattern extended to awareness and engagement with DEI initiatives, where administrators and faculty demonstrated higher levels of awareness compared to other institutional roles.

A similar hierarchical pattern emerged in belonging measures, with administrators holding faculty positions reporting the strongest sense of belonging (mean = 4.10, SD = 0.787), while staff members indicated the lowest scores (mean = 3.58, SD = 0.939). Faculty and student scores fell between these extremes, suggesting a systematic relationship between institutional role and perceived connection to the educational community.

Well‐being measures reinforced these role‐based patterns across personal, social, and climate‐related dimensions. Administrators with faculty roles consistently reported the highest scores (personal: mean = 4.01, SD = 0.778; social: mean = 4.11, SD = 0.638; and climate‐related: mean = 4.17, SD = 0.777), while staff and students reported lower scores across all well‐being measures. These consistent patterns across all three climate dimensions suggest that institutional role substantially influences overall experience within the educational environment (complete results presented in Tables 2, 3, 4).

**TABLE 2:** Responses concerning feeling welcome by type of respondent.

Statements related to feeling welcome	Students (*N* = 8485), mean (SD)	Staff (*N* = 1994), mean (SD)	Faculty (*N* = 3393), mean (SD)	Admin (*N* = 293), mean (SD)	Admin and faculty (*N* = 395), mean (SD)	*p*‐value
**Individual item**						
49. The climate is welcoming to individuals from diverse backgrounds and experiences.	4.06^a^ (0.924)	3.99 (0.930)	4.19 (0.970)	4.08 (0.872)	4.26 (0.839)	<0.001

All respondents: my dental school/allied dental education program …	Students	Staff	Faculty	Admin	Admin and faculty	*p*‐value
18. … fosters a sense of community.	3.81 (1.001)	3.54 (1.054)	3.81 (1.082)	3.81 (1.014)	3.97 (0.877)	<0.001
19. … has a strong sense of social cohesion.	3.69 (1.045)	3.38 (1.061)	3.57 (1.141)	3.53 (1.062)	3.71 (0.931)	<0.001
20. … values students’ opinions.	3.39 (1.257)	3.69 (0.980)	4.05 (0.961)	4.03 (0.827)	4.27 (0.785)	<0.001
“All respondents feel welcome” index^b^ (Cronbach alpha = 0.858)	3.63 (0.987)	3.54 (0.924)	3.81 (0.949)	3.79 (0.856)	3.98 (0.731)	<0.001

Students feeling welcome	Students	Staff	Faculty	Admin	Admin and faculty	*p*‐value
36. Students know that they can get assistance from faculty if they need help.	3.97 (0.973)	n/a	n/a	n/a	n/a	–
37. Students know that they can get assistance from staff if they need help.	3.95 (0.964)	n/a	n/a	n/a	n/a	–
38. Students know that they can get assistance from other students if they need help.	4.25 (0.780)	n/a	n/a	n/a	n/a	–
“Students feel welcome” index^c^ (Cronbach alpha = 0.827)	4.06 (0.785)	n/a	n/a	n/a	n/a	***–***

My dental school/allied dental education program has …	Students, % yes	Staff, % yes	Faculty, % yes	Admin, % yes	Admin and faculty, % yes	*p*‐value
53. A DEI office or similar office/department.	51.4%	64.3%	69.3%	77.6%	75.9%	<0.001
54. A dedicated DEI officer or similar leadership position.	42.5%	56.4%	62.8%	73.5%	74.6%	<0.001
55. A DEI committee comprised of individuals representing students, faculty, etc.	38.3%	46.7%	50.7%	64.7%	69.9%	<0.001
“DEI committee welcome” sum score^d^	2.64 (0.908)	2.64 (0.850)	2.68 (0.829)	2.60 (0.863)	2.49 (0.946)	0.012

Abbreviations: Admin, Administrators; DEI, diversity, equity, and inclusion; n/a, not available; SD, standard deviation.

^a^ Answers ranged from 1 = “Strongly Disagree”, 2 = “Disagree”, 3 = “Neither Agree nor Disagree”, 4 = “Agree” to 5 = “Strongly Agree”.

^b^ The “All respondents feel welcome” Index was computed by averaging the responses to questions 18–20.

^c^ The “Students feel welcome” Index was computed by averaging the responses to questions 36–38.

^d^ “DEI committee welcome” Sum score was computed by adding one point for each “Yes” response to questions 53–55.

**TABLE 3:** Answers to questions assessing a sense of belonging by type of respondent.

No sense of belonging: at my dental school/allied dental education program	Students, % yes	Staff, % yes	Faculty, % yes	Admin, % yes	Admin and faculty, % yes	*p*‐value
92. … I have witnessed discrimination.	21.7%	27.8%	23.6%	25.7%	34.6%	<0.001
93. … I have experienced discrimination.	11.9%	16.8%	17.1%	13.4%	18.1%	<0.001
94. … I have witnessed harassment.	14.6%	22.2%	19.3%	26.5%	31.8%	<0.001
95. … I have experienced harassment.	8.7%	17.4%	14.3%	16.8%	17.5%	<0.001
“No sense of belonging” sum score^a^	0.54 (1.045)	0.79 (1.291)	0.70 (1.22)	0.78 (1.226)	1.00 (1.332)	<0.001

Having a sense of belonging: at my dental school/allied dental education program	Students, mean (SD)	Staff, mean (SD)	Faculty, mean (SD)	Admin, mean (SD)	Admin and faculty, mean (SD)	*p*‐value
51. Members of the community come together to celebrate good and share difficult times.	3.93^b^ (0.959)	3.58 (1.050)	3.77 (1.078)	3.84 (0.950)	3.93 (0.998)	<0.001
52. Members of my community care about each other.	3.99 (0.921)	3.69 (0.995)	3.99 (0.981)	4.00 (0.874)	4.15 (0.867)	<0.001
12. We are a community where we look out for each other.	3.82 (1.038)	3.47 (1.113)	3.76 (1.134)	3.82 (1.000)	3.98 (0.964)	<0.001
13. I feel a sense of belonging.	3.78 (1.069)	3.52 (1.123)	3.89 (1.132)	3.92 (1.007)	4.11 (0.972)	<0.001
14. I feel like I am a member of my community.	3.87 (0.996)	3.54 (1.093)	3.94 (1.075)	3.99 (0.948)	4.21 (0.882)	<0.001
16. I would choose my dental school/allied dental education program over again.	3.75 (1.177)	3.63 (1.015)	4.01 (1.083)	3.97 (0.902)	4.18 (0.942)	<0.001
17. is supportive of me.	3.82 (1.049)	3.57 (1.068)	3.88 (1.114)	3.95 (0.941)	4.09 (0.922)	<0.001
“Sense of belonging” index^d^ (Cronbach alpha = 0.941)	3.86 (0.872)	3.58 (0.939)	3.90 (0.949)	3.94 (0.820)	4.10 (0.787)	<0.001

Abbreviation: Admin, Administrators; SD, standard deviation.

^a^ The “No sense of belonging” Sum score was computed by adding one point for each “Yes” response to questions 92–95.

^b^ Answers ranged from 1 = “Strongly Disagree”, 2 = “Disagree”, 3 = “Neither Agree nor Disagree”, 4 = “Agree” to 5 = “Strongly Agree”.

^c^ The “Sense of belonging” Index was computed by averaging the responses to questions 51, 52, 12 14 and 16 and 17.

**TABLE 4:** Responses concerning having a sense of well‐being by respondent type.

	Students, mean (SD)	Staff, mean (SD)	Faculty, mean (SD)	Admin, mean (SD)	Admin and faculty, mean (SD)	*p*‐value
Personal well‐being: … dental school/allied dental education program.						
9. I am satisfied with the over‐all climate of my …	3.64^a^ (1.112)	3.41 (1.128)	3.64 (1.207)	3.58 (1.088)	3.66 (1.088)	<0.001
15. I feel comfortable at my …	3.89 (1.009)	3.71 (0.998)	4.01 (1.037)	4.09 0.881	4.19 (0.833)	<0.001
22. I am comfortable being my authentic self at my …	3.82 (1.042)	3.66 (1.045)	3.87 (1.098)	3.90 0.997	4.05 (0.914)	<0.001
23. I am respected within my … culture.	3.90 (0.954)	3.68 (0.990)	4.03 (0.963)	4.03 0.891	4.13 (0.887)	<0.001
“Personal well‐being” index^b^ (Cronbach alpha = 0.892)	3.82 (0.897)	3.62 (0.901)	3.89 (0.941)	3.91 (0.818)	4.01 (0.778)	<0.001

Social well‐being: I have the opportunity to develop trusting relationships	Students	Staff	Faculty	Admin	Admin and faculty	*p*‐value
24. …with students.	4.16 (0.805)	3.78 (0.923)	4.28 (0.753)	3.92 (0.806)	4.30 (0.680)	<0.001
25. … with faculty.	3.90 (0.967)	3.75 (0.981)	4.07 (0.942)	4.06 (0.838)	4.25 (0.739)	<0.001
26. …with staff.	3.90 (0.939)	3.91 (0.915)	4.11 (0.869)	4.14 (0.772)	4.27 (0.720)	<0.001
27. I have not experienced intimidation at my …	3.39 (1.292)	3.48 (1.257)	3.73 (1.310)	3.65 (1.265)	3.74 (1.237)	<0.001
45. I am satisfied with the present state of relationships between members of my …	3.85 (0.982)	3.55 (0.995)	3.80 (1.074)	3.69 (0.963)	3.85 (0.979)	<0.001
50. I enjoy spending time with members of my …	4.08 (0.875)	3.84 (0.922)	4.15 (0.892)	4.09 (0.866)	4.24 (0.856)	<0.001
“Social well‐being” index^c^ (Cronbach alpha = 0.868)	3.88 (0.767)	3.72 (0.778)	4.02 (0.778)	3.93 (0.677)	4.11 (0.638)	<0.001

Climate‐related well‐being	Students	Staff	Faculty	Admin	Admin and faculty	*p*‐value
10a. Student health and well‐being are a priority at my …	3.47 (1.254)	3.87 (0.970)	4.17 (0.951)	4.09 (0.916)	4.30 (0.808)	<0.001
11. The climate in my … encourages free and open discussion about students’ health and well‐being.	3.57 (1.152)	3.57 (1.000)	3.84 (1.066)	3.92 (0.922)	4.04 (0.898)	<0.001
“Climate‐related well‐being” index^d^ (Cronbach alpha = 0.865)	3.52 (1.143)	3.72 (0.907)	4.01 (0.929)	4.00 (0.842)	4.17 (0.777)	
Climate related well‐being						
10b. Faculty health and well‐being are a priority at my …	n/a	a/a	3.52 (1.273)	n/a	n/a	–
10c. Staff health and well‐being are a priority at my …	n/a	3.06 (1.275)	n/a	3.28 (1.277)	3.59 (1.213)	<0.001

Abbreviation: Admin, Administrators; n/a, not available; SD, standard deviation.

^a^ Answers ranged from 1 = “Strongly Disagree”, 2 = “Disagree”, 3 = “Neither Agree nor Disagree”, 4 = “Agree” to 5 = “Strongly Agree”.

^b^ The “Personal well‐being” Index was computed by averaging the responses to questions 9, 15, 22 and 23.

^c^ The “Social well‐being” Index was computed by averaging the responses to questions 24 to 26 and 45 and 50.

^d^ The “Climate‐related well‐being” Index was computed by averaging the responses to questions 10a and 11.

### 3.2 Relationships between demographics and climate perceptions

Our examination of demographic factors revealed several significant correlations that provide additional context for understanding climate perceptions. Age showed strong positive correlations with years in dental education (*r* = 0.64, *p* < 0.001) and years in current employment (*r* = 0.52, *p* < 0.001). However, the relationship between age and climate perceptions proved more nuanced. We found only minimal associations between age and climate measures, including a very weak negative correlation with welcoming climate perception (*r* = ‒0.01), a weak positive correlation with the “all welcome” index (*r* = 0.05, *p* < 0.001), and a weak negative correlation with the “student welcome” index (*r* = ‒0.05, *p* < 0.001). These findings suggest that while professional experience tends to accumulate with age, perceptions of institutional climate may be influenced more by role and position than by demographic factors alone (complete correlational analyses presented in Table 5).

**TABLE 5:** Correlations between climate indices and demographics.⁵

	Age	A. Years in education	B. Years in current work	C.^a^ Climate is welcoming	D.^b^ “All welcome” index	E.^b^ “Student welcome” index	F.^b^ Welcome sum score
Age and years in dental education and in current employment							
Age	1	0.64^***^	0.52^***^	‒0.01	0.05^***^	‒0.05^***^	‒0.01
A. Faculty/staff: years in dental education	0.64^***^	1	0.66^***^	‒0.02	0.01	n/a^5^	‒0.03
B. Faculty/staff: years in current employment	0.52^***^	0.66^***^	1	‒0.05	‒0.04	n/a^5^	‒0.06

Welcome	Year of birth	A	B	C	E	D	F
C. The climate is welcoming.^a^	‒0.01	‒0.02	‒0.05	1	0.76^***^	0.63^***^	0.29^***^
D. “All respondents feel welcome” index	0.05^***^	‒0.01	‒0.04	0.76^***^	1	0.71^***^	0.30^***^
E. “Students feel welcome” index	‒0.05^***^	n/a	n/a	0.63^***^	0.71^***^	1	0.30^***^
F. “DEI committee welcome” sum score	‒0.01	‒0.03	‒0.06	0.29^***^	0.30^***^	0.30^***^	1

Belonging	Year of birth	A	B	C	E	D	F
“No sense of belonging” sum score^c^	0.07^***^	0.04	0.08^****^	‒0.50^***^	‒0.48^***^	‒0.39^***^	‒0.23^***^
“Sense of belonging” index	0.01	0.01	‒0.01	0.79^***^	0.86^***^	0.74^***^	0.30^***^

Well‐being	Year of birth	A	B	C	E	D	F
“Personal well‐being” index^d^	0.03	0.02	‒0.01	0.86^***^	0.83^***^	0.73^***^	0.30^***^
“Social well‐being” index^d^	‒0.06^***^	‒0.03	0.01	0.73^***^	0.81^***^	0.78^***^	0.32^***^
Climate‐related well‐being” index^d^	‒0.17^***^	‒0.01	0.02	0.70^***^	0.78^***^	0.64^***^	0.29^***^
Nr10b. Faculty health and well‐being^a^	‒0.02	‒0.00	0.08^***^	0.52^***^	0.73^***^	n/a	0.19^***^
Nr10c. Staff health and well‐being^a^	‒0.11^***^	‒0.06	‒0.02	0.45^***^	0.65^***^	n/a	0.22^***^

*Note*: * p<0.05; ** p<0.01; *** p <0.001

Abbreviation: DEI, diversity, equity, and inclusion; n/a, not available.

^a^ The answers ranged from 1 = “disagree strongly”, 2 = “disagree”, 3 = “neither disagree nor agree”, 4 = “agree” to 5 = “agree strongly”.

^b^ The legend of Table 2 explains how the “All respondents feel welcome” Index, the “Students feel welcome” Index and the “DEI committee welcome” Sum score were constructed.

^c^ The legend of Table 3 explains how the “No sense of belonging” Sum score And the “Sense of belonging” Index were constructed.

^d^ The legend of Table 4 explains how the “Personal well‐being” Index, the “Social well‐being” Index and the “Climate‐related well‐being” Index were constructed.

These findings provide important insights into the varied experiences of different groups within dental education institutions, revealing patterns that merit deeper examination. Below, we discuss the implications of these role‐based disparities and their significance for creating more inclusive educational environments, connecting our findings to existing literature and practical applications.

## 4 DISCUSSION

Our analysis of the ADEA's Climate Study data reveals compelling patterns in how different groups experience the dental education environment, with significant implications for institutional policy and practice. The data demonstrate systematic disparities in perceptions of welcomeness, belonging, and well‐being across institutional roles, suggesting that one's position within the educational hierarchy substantially influences their experience of the institutional climate.

### 4.1 Role‐based disparities in climate perception

A striking pattern emerged in our analysis: administrators and faculty consistently reported more positive experiences compared to students and staff across all measured dimensions. This hierarchical stratification of experience likely reflects deeply embedded institutional structures, including power dynamics, differential access to resources, and varying levels of agency within the organization. These findings align with Ester et al.’s research, which identified similar role‐based disparities in dental education settings. The consistency of these patterns suggests that these differences are not merely coincidental but rather reflect systemic aspects of how dental education institutions operate.

### 4.2 Institutional climate and welcomeness

The concept of welcomeness in educational institutions is pivotal to the formation of a sense of connectedness, both socially and institutionally, from the program's beginning, in order to create an inclusive and supportive environment where all members feel valued and respected.^5^ The analysis revealed noteworthy variations in perceptions of welcomeness across institutional roles. Faculty and administrators reported significantly higher welcomeness scores (mean = 4.19/5.0) compared to students (4.06/5.0) and staff (3.99/5.0). These differences, while seemingly modest in absolute terms, point to meaningful variations in how different groups experience the institutional environment. Didion et al.’s framework provides valuable context for these findings, suggesting that institutional practices significantly influence constituent satisfaction and engagement.^6^ ^,^ ^7^ Particularly telling was the disparity in awareness of DEI initiatives between leadership and other groups, indicating a potential communication gap that merits institutional attention.

### 4.3 Complex dynamics of belonging

A sense of belonging in educational settings refers to the feeling of being accepted, valued, and included within a community. Researchers characterized belongingness as an indicator of valuing and embracing one's diversity and conveying welcomeness.^8^, ^9^, ^10^ Perhaps our most compelling finding was the marked disparity in belonging scores between administrators with faculty roles (mean = 4.10/5.0) and staff members (mean = 3.58/5.0). This substantial difference extends Radford and Hellyer's research on belongingness in professional development by demonstrating how institutional role significantly influences one's sense of connection to the academic community.^11^ Additionally, Fleming et al. addressed the challenges faced by Black faculty members, underscoring the need for anti‐racist practices and supportive environments to combat racial battle fatigue and promote belonging.^12^ The consistently lower scores among staff members suggest that traditional institutional structures may inadvertently marginalize certain groups, even as they seek to create inclusive environments.^13^ ^,^ ^14^ Vivekananda‐Schmidt and Sandars further highlight the impact of belongingness on student motivation, learning identity, and mental health.^15^

### 4.4 Well‐being through an institutional lens

Well‐being in educational settings refers to the overall physical, mental, and emotional health of individuals within the institution. The systematic variation in well‐being measures across roles provides new insights into how institutional position influences personal and professional flourishing. Administrators with faculty roles consistently reported higher scores across all well‐being dimensions (personal: 4.01/5.0; social: 4.11/5.0; and climate‐related: 4.17/5.0). These findings build upon Chattu et al.’s work on well‐being in health professions education by demonstrating how institutional role mediates individual experience.^16^ The consistent pattern suggests that institutional position significantly influences not just professional satisfaction but overall well‐being within the educational environment.

Well‐being is crucial for overall student success and mental health, as it directly influences engagement, academic performance, and personal development.^16^ Ensuring a high level of well‐being helps students and staff to thrive in their educational and professional roles, fostering a positive and productive learning environment.^17^, ^18^, ^19^, ^20^, ^21^ Research indicates that well‐being is closely linked to a sense of belonging and feeling welcome within the educational environment. Studies by Radford and Hellyer and Vivekananda‐Schmidt and Sandars emphasize that a strong sense of belonging enhances motivation, identity formation, and mental health.^2^ ^,^ ^15^

### 4.5 Challenging traditional assumptions

Our analysis of demographic factors yielded unexpected results that challenge conventional wisdom about institutional climate. The minimal impact of age and experience on climate perceptions contradicts traditional assumptions about seniority and satisfaction. These findings suggest that positive climate experiences are more closely tied to institutional role and structure than to individual characteristics or length of service, pointing to the need for more nuanced approaches to fostering inclusion.

### 4.6 Implications for institutional practice

These findings highlight several critical areas requiring institutional attention. First, the consistently lower scores among staff across all measures suggest a need for targeted interventions to improve their experience. Second, the awareness gap regarding DEI initiatives between leadership and other groups indicates a need for more effective communication strategies. Third, the strong relationship between institutional role and climate experience suggests that structural changes may be necessary to create more equitable environments.

### 4.7 Future directions

This analysis opens several promising avenues for future research. Longitudinal studies tracking changes in climate perceptions over time could provide valuable insights into the effectiveness of interventions. Qualitative investigations could deepen our understanding of role‐based experience differences, while studies examining the intersection of institutional roles with other demographic factors could reveal more complex patterns of experience. Additionally, evaluation of specific intervention strategies could help identify effective approaches to improving institutional climate for all members of the dental education community.

Our analysis reveals important insights about institutional climate in dental education, particularly how different roles experience the educational environment. The consistent pattern of role‐based disparities, combined with the minimal impact of age and experience, suggests that structural factors rather than individual characteristics drive climate perceptions. These findings point to specific areas for intervention and improvement in creating more inclusive dental education environments.

### 4.8 Recommendations for institutional practice

Our analysis of the ADEA's Climate Study data, combined with insights from current literature, points to several actionable recommendations for improving institutional climate in dental education. The findings directly suggest the need for targeted support initiatives for staff members, who consistently reported lower scores across all climate measures. Addressing the observed gaps in awareness and engagement requires enhanced communication channels between leadership and other institutional roles. This could be accomplished through specific structural changes: implementing regular cross‐role climate forums, establishing structured feedback mechanisms for staff and students, and creating transparent reporting systems for climate initiatives and outcomes.^22^ ^–^ ^24^ Such communication structures would facilitate better information flow while promoting more inclusive institutional dialog.

Previous research provides additional support for specific interventions. Campbell et al.^25^ demonstrate the value of prioritizing DEIB officer positions. Other studies recommend establishing active committees to guide institutional climate initiatives.^26^ ^,^ ^27^ The importance of mentorship and coaching programs, particularly for staff and junior faculty members.^28^ ^,^ ^29^ Haim‐Litevsky et al. found such programs significantly improve institutional connection and professional development.^30^ Piano et al. further contribute specific strategies for enhancing diversity and inclusion in health sciences education, emphasizing the importance of structured programs that increase engagement across all institutional roles.^31^

These recommendations, derived from both our empirical findings and supporting literature, provide a framework for improving institutional climate in dental education. By focusing on role‐specific experiences while maintaining a holistic view of institutional climate, these suggestions offer practical approaches to creating more inclusive and supportive educational environments for all members of the dental education community.^32^ ^,^ ^33^

### 4.9 Study limitations

While our analysis provides valuable insights into institutional climate in dental education, several important limitations warrant consideration. First, the scope of our analysis focused specifically on role‐based differences, despite the ADEA Climate Study's collection of broader institutional data. While this focus allowed for detailed examination of role‐based variations, it necessarily excluded analysis of program types (dental school vs. allied dental programs), geographic locations, and institutional characteristics (public/private). These factors likely influence climate perceptions and represent important areas for future investigation.

The structure of our data presents additional limitations. The cross‐sectional nature of the study provides a snapshot of institutional climate but cannot capture changes over time or the dynamic nature of institutional environments. While the survey included both quantitative and qualitative components, our analysis focused exclusively on quantitative measures to maintain analytical focus and feasibility.^34^ This approach, while providing robust statistical findings, may not capture the full complexity of individual experiences within dental education institutions.

Response patterns present another important consideration. Despite the large sample size (*n* = 14,560), participation rates likely varied across institutions and roles. The voluntary nature of survey participation introduces potential self‐selection bias, raising the possibility that the experiences of non‐respondents may differ systematically from those who chose to participate. This limitation particularly affects our ability to generalize findings to the broader dental education community.

Finally, measurement considerations affect the interpretation of our findings. The indices used in this analysis, while carefully constructed, represent only one approach to measuring these complex constructs. The relationships we observed between measured variables may be influenced by unmeasured factors, and alternative approaches to measuring institutional climate might yield additional insights.

These limitations, while important to acknowledge, do not diminish the significant contributions of this analysis to understanding institutional climate in dental education. Rather, they suggest valuable directions for future research, including:

- Examination of institutional characteristics' influence on climate perceptions.
- Longitudinal studies tracking changes in climate measures over time.
- Mixed‐methods approaches incorporating qualitative data to provide richer context.
- Investigation of alternative measurement approaches for institutional climate constructs.

Our findings provide a foundation for understanding role‐based variations in institutional climate while acknowledging the need for continued research to fully understand this complex phenomenon.

## 5 CONCLUSION

The study's findings underscore the critical importance of creating supportive and inclusive environments that enhance welcomeness, sense of belonging, and well‐being for all members of the dental education community. By acknowledging and addressing the disparities in perceptions reported by various stakeholders, dental education programs can implement evidence‐based strategies to foster a more cohesive and supportive climate. The data reveal that targeted approaches to professional development, mentorship, and community‐building initiatives can significantly impact individual experiences and institutional outcomes. This focus on improving perceptions of welcomeness, sense of belonging, and well‐being serves a dual purpose: it enhances the personal and professional growth of individuals while strengthening the foundation for academic excellence and innovation within educational institutions. As dental education continues to evolve, the insights gained from this study provide a framework for developing and implementing practices that promote both individual success and institutional advancement.

## DISCLAIMER

This article is based on empirical research. The analysis and interpretations expressed in this piece are solely those of the authors and not intended to represent or reflect the position or viewpoints of the American Dental Education Association, its publisher, or any entities with whom the authors may or may not be affiliated or associated.

[Correction added on 27 May 2025, after first online publication: A disclaimer section has been added to this version.]
